# Dynamic Changes of Fecal Calprotectin and Related Clinical Factors in Neonates

**DOI:** 10.3389/fped.2020.00326

**Published:** 2020-07-08

**Authors:** Ji Sook Park, Jae Young Cho, Changyeong Chung, Seong Hee Oh, Hyun-jeong Do, Ji-Hyun Seo, Jae Young Lim, Chan-Hoo Park, Hyang-Ok Woo, Hee-Shang Youn

**Affiliations:** ^1^Department of Pediatrics, College of Medicine, Gyeongsang National University, Jinju, South Korea; ^2^Department of Pediatrics, Gyeongsang National University Hospital, Jinju, South Korea; ^3^Institute of Health Sciences, Gyeongsang National University, Jinju, South Korea; ^4^Department of Pediatrics, Gyeongsang National University Changwon Hospital, Changwon, South Korea

**Keywords:** calprotectin, neonate, meconium, breast milk, postnatal week

## Abstract

**Objective:** Fecal calprotectin (FC) has been widely used for a clinical marker of intestinal inflammation in children and adults. However, the clinical usefulness has not been determined in neonates. The purpose of this study was to investigate the change of FC and associated clinical factors in neonates.

**Methods and Materials:** In total, 146 neonates among 472 admissions to our NICU between 2018 and 2019 were included, and 242 stool samples were collected. FC was measured in the first, second, and third–fourth week after birth, respectively, using commercial ELISA. The clinical characteristics were reviewed from medical records. Statistical analyses were performed to analyze associated factors regarding on changes of fecal calprotectin.

**Results:** A wide range from 5.5 to 6,000 mg/kg of FC was observed in neonates. FCs during neonatal period were not correlated with the gestational age at birth or birth weight. The meconial calprotectin was higher than FCs after 2 weeks of age (*n* = 134, 418.06 vs. 243.12 in the second week and 259.58 in the third week after birth). Meconial calprotectin was associated with birth weight and meconium stained amniotic fluid. FC during the neonatal period decreased with postnatal week (−464.93 ± 158.02 at third–fourth week after birth compared with the 1st week, *P* = 0.004) and breast milk (−337.27 ± 150.51 compared with formula milk, *P* = 0.026).

**Conclusion:** Fecal calprotectin tended to decrease with postnatal week during the neonatal period, and breast milk could affect more decrease of FC.

## Introduction

Calprotectin is a 36.5-kDa calcium- and zinc-binding protein found mostly in neutrophils, monocytes, and macrophages and involved in the regulation of inflammation (1, 2). This protein is stable in the feces for 1 week at room temperature, and commercial enzyme-linked immunosorbent assay (ELISA) kits are available for extraction and analysis of small stool samples, ~0.1 g of stool. Fecal calprotectin (FC) concentration is directly proportional to neutrophil migration toward the gastrointestinal tract and closely correlates with leukocyte excretion in the feces (3). FC has been studied as a noninvasive and useful marker for bowel inflammation in children and adults such as inflammatory bowel disease and other organic intestinal and allergic disorders (3–8). For adults and children, <50 μg/g of FC in stool has been considered within normal limits (9). However, FC concentration has age-dependent variation in children, especially those younger than 4 years, and increases in infants (10, 11). Normally, a high level of FC was reported in healthy neonates, and the usefulness of this marker remains controversial in the neonatal period (2). Although the clinical usefulness of FC in newborns has been studied in diverse aspects including gestational period, birth weight (BW), delivery modes, diet, antibiotic treatment, necrotizing enterocolitis (NEC), or gut microbiota, it has been inconclusive because of high interindividual variations, inhomogeneous subjects, and different sampling times according to previous studies (12–16). Yang et al. (17) reported that serial changes of FC could be affected by minor or major systemic and gastrointestinal stress in 14 very-low-birth-weight infants. However, Zoonen et al. (18) reported that serial FCs could not predict NEC in 40 preterm infants including 10 infants with NEC and 30 controls. There is still controversy on the clinical meaning of FC and serial changes of FC in preterm and term neonates. Thus, the purpose of this study was to investigate associating clinical factors with serial changes of FC during the neonatal period.

## Method

### Subjects and Stool Collection

Among 472 admissions at the neonatal intensive care unit (NICU) in our hospital between May 2018 and October 2019, stool samples for FC were collected prospectively from 146 neonates born at 25–40 weeks of gestation, with parent's consent. In total, 242 stool samples were collected, with 134 in the first, 67 in the second, and 41 in the third–fourth weeks of life. Stool samples were stored at 4°C without preservative and measured within 5 days of collection using commercial ELISA (Calprolab™ Calprotectin ELISA, Calpro AS, Norway) following the manufacturer's instruction. Two or more serial stools were collected from 64 among 146 neonates.

### Clinical Data Collection

The clinical characteristics were retrospectively reviewed from medical records. The prenatal and perinatal clinical factors were investigated as follows.

(1) Multiple gestation such as two or more fetuses in one pregnancy.
(2) Meconium-stained amniotic fluid (AF) such as meconium pass before giving birth.
(3) Prolonged premature rupture of membrane (PPROM) such as rupture of amniotic membrane over 18 h before birth.
(4) Maternal hypertensive condition such as essential hypertension, pregnancy-induced hypertension (PIH), eclampsia, or preeclampsia.
(5) Respiratory distress syndrome (RDS) of newborn such as ground glass opacity of lungs on chest X-ray with tachypnea and/or desaturation.
(6) Use of antibiotics such as any kind of antibiotic treatment for suspicious or culture-proven sepsis during the neonatal period.
(7) Neonatal jaundice such as unconjugated hyperbilirubinemia that needs phototherapy in preterm and term neonates (19).
(8) Intestinal distress such as gastrointestinal symptoms or signs leading to interrupted enteral feeding and suspected or definite NEC based on modified Bell's classification (20).
(9) Days of full feeding such as postnatal days until fed enterally with 120 ml/kg/day.
(10) Breast milk (BM) feeding such as being fed with BM exclusively or partially with BM being >1/2 of the total amount of feeding volume.

### Statistical Analysis

Statistical analyses were conducted using SPSS® version 25.0 (IBM, NY, US), and GraphPad Prism 6 (Graph-Pad Software, CA, USA) was used for graphics. Normality of the continuous values was tested by Kolmogorov–Smirnov and Shapiro–Wilk tests. The continuous variables were presented as the mean and standard deviation (SD) or median and interquartile range (IQR). Comparisons of FC according to clinical factors were performed by independent *t*-test or the Mann–Whitney *U*-test. Correlation between FC and continuous variables was performed by the Spearman rho test. The categorical data were presented as the number and proportion (%). Multivariable linear or nonlinear logistic regression analysis was conducted to investigate an association between clinical factors and the level of FC. We also checked the multicollinearity between the factors for adjustment using correlation coefficients and excluded one when the coefficient was more than 0.7. The changes in FC concentration according to clinical factors during the neonatal period were investigated using a linear mixed model (LMM) or generalized linear mixed-effect model (GLMM), a repeated-measures covariance pattern model with unstructured covariance within the subjects. The *P*-value was set as <0.05.

### Ethics Statement

The protocol of this study was reviewed and approved by the institutional review board of our hospital (GNUH 2020-02-004). Informed consent was waived by the board.

## Results

### Clinical Characteristics

The clinical characteristics were described in Table 1. In total, 146 neonates were enrolled and born at a mean of 34.6 ± 3.5 weeks of gestation (range: 25^4/7^-40^3/7^). Among them, preterm neonates born before 37 weeks of gestation were 109 (74.7%). Mean BW was 2.2 ± 0.8 kg (range: 0.6–4.3), and low-birth-weight infants weighing less than 2.5 kg were 93 (63.7%). Twenty-eight (19.2%) were born vaginally. Males were 68 (46.6%). Mean Apgar score at 5 min was 8.8 ± 1.3. Obstetric problems occurred in 89 pregnancies, which were maternal hypertensive disorder (24.7%, 36 of 146 neonates), gestational diabetes mellitus (GDM, 15.1%, 22 of 146 neonates), PPROM (11.0%, 16 of 146 neonates), and meconium pass *in utero* (10.3%, 15 of 146 neonates) in that order. Forty-three neonates (29.5%) among 146 neonates were born as twins or triplets.

**Table 1:** Clinical characteristics and level of fecal calprotectin in total subjects (*N* = 146).

**Variables**	**Mean ± SD, *N* (%)**	**Median (IQR)**	**Min, Max**
**Prenatal variables**			
Multiple gestation	43 (29.5)		
Meconium-stained AF	15 (10.3)		
Maternal hypertensive disorder	36 (24.7)		
PPROM	16 (11.0)		
GDM	22 (15.1)		
**Perinatal variables**			
Gestational age, weeks	34.56 ± 3.47	35.00 (32.25, 37.04)	25.57, 40.43
Birth weight, kg	2.21 ± 0.82	2.20 (1.56, 2.76)	0.60, 4.31
Male	68 (46.6)		
5′-AS	8.79 ± 1.34	9.00 (8.00, 10.00)	3, 10
Vaginal delivery	28 (19.2)		
**Postnatal variables**			
RDS	54 (37.0)		
Jaundice	27 (18.5)		
Use of antibiotics	95 (65.1)		
Intestinal distress	17 (11.6)		
Breast milk	110 (75.3)		
Days till full feeding (120 ml/kg)	9.67 ± 12.02	5.0 (3.0, 10.0)	1, 73
Hospital stays, day	26.34 ± 25.93	16.0 (9.0, 35.0)	2, 156
Weight at discharge, kg	2.77 ± 0.61	2.66 (2.37, 3.11)	1.08, 5.84
Mortality	2 (1.4)		
Fecal calprotectin, mg/kg stool	341.68 ± 687.39	154.65 (70.48, 346.03)	5.50, >6,000
Week 1 (*n* = 134)	418.06 ± 864.89	197.75 (74.05, 394.68)	6.10, >6,000
Week 2 (*n* = 67)	243.12 ± 328.80	123.00 (63.20, 292.50)	5.50, 1,792.0
Weeks 3–4 (*n* = 41)	259.58 ± 368.18	162.25 (88.55, 248.23)	20.6, 2,061.0

*SD, standard deviation; IQR, interquartile range; meconium-stained AF, meconium-stained amniotic fluid; PPROM, prolonged rupture of membrane; GDM, gestational diabetes mellitus; 5′-AS, Apgar score at 5 min; RDS, respiratory distress syndrome; jaundice, unconjugated hyperbilirubinemia requiring phototherapy*.

Chief complaints at admission among 146 neonates were prematurity (59.6%), maternal condition affecting baby's health (17.5%), and low BW or being small for gestation (13.8%), in that order. Rates of BM feeding was 75.3% (110 of 146 neonates), and mean time to achieve full feeding with enteral route was 9.7 ± 12.0 days. Intestinal distress leading to the interruption of enteral or oral feeding including NEC, occurred in 17 of 146 neonates (11.6%). Mean hospital stays were 26.3 ± 25.9 days and weighed mean 2.8 ± 0.6 kg at discharge from the NICU. Two infants died from sepsis at the 24th and 74th days after birth.

### Concentration of FC

**Figure 1:**
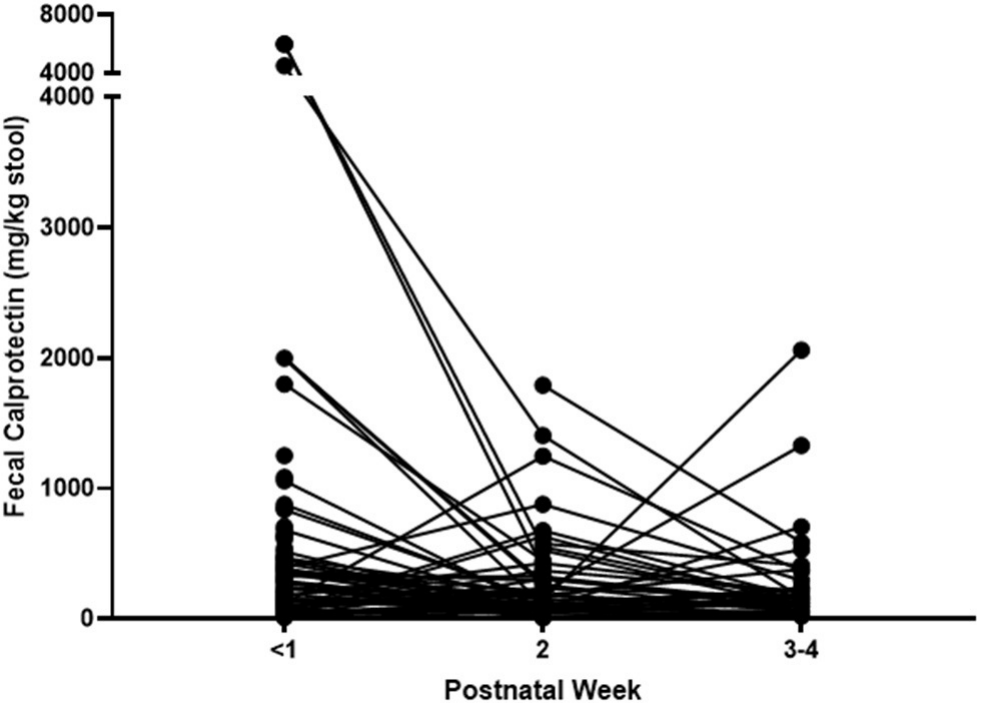
Changes of fecal calprotectin of individual subjects (*N* = 146).

FCs were measured in the first, second, and third–fourth weeks after birth, and a total of 242 FCs were measured from 146 neonates. The FC of the first week was mean 418.06 ± 864.89 mg/kg of stool and was collected from 134 neonates on mean day of 2.14 ± 1.87 after birth (median 1.0, IQR: 1.0–3.0). The second-week FC was mean 243.12 ± 328.80 mg/kg of stool and was collected from 67 neonates on mean day of 10.03 ± 0.43 after birth (median 9.0, IQR: 8.0–12.0). The third–fourth week FC was mean 259.58 ± 368.18 mg/kg of stool and was collected on mean day of 21.29 ± 1.07 after birth (median 21.0, IQR: 15.8–24.0). The lowest level of FC was 5.5, and the highest one was over 6,000 mg/kg. Changes of FC in individual subject are shown in Figure 1.

The mean concentration of FC during neonatal period was 341.68 ± 687.39 mg/kg, in total. FCs were not correlated with the gestational age (GA, *r* = −0.125, *P* = 0.151) or BW (*r* = −0.144, *P* = 0.097).

### Associating Factor With High Meconial Calprotectin Concentration

**Figure 2:**
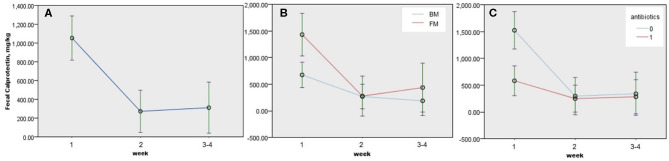
Estimated changes and 95% confidence interval (CI) of fecal calprotectin (FC) in neonates according to the postnatal week and clinical factors among 64 neonates with two or more consecutive FCs. **(A)** Time trend of FC during the neonatal period (*P* < 0.005), **(B)** time trends of FC according to diet during the neonatal period (*P* = 0.026), and **(C)** time trends of FC according to antibiotic treatment (*P* = 0.004). *P*-values were obtained by GLMM, and continuous predictors were fixed at 1.68 kg of birth weight. BM, breast milk; FM, formula milk.

The first-week FC was measured by meconium since feces were collected at mean 2.14 ± 1.87 days after birth (median 1.0 day, IQR: 1.0–3.0). Meconial calprotectin was higher than later FCs (Table 1, Figures 1, 2). Meconial calprotectin was higher in females (626.43 ± 1,131.04 mg/kg) than in males (175.85 ± 178.03 mg/kg, *P* < 0.001). We performed stepwise multivariable linear or nonlinear regression analysis to investigate associating prenatal or perinatal factors with meconial calprotectin. Of them, lower BW (−284.25 ± 91.88 mg/kg per kilogram increase, *P* = 0.002) and meconium-stained AF (464.70 ± 232.14 mg/kg, *P* = 0.047) were associated with high concentration of meconial calprotectin (Table 2). GA and gender were not considered as associating factors with meconial calprotectin because GA had high collinearity with BW (*r* = 0.872, *P* < 0.001) and BW was different according to gender in this study (females: 2.09 ± 0.78 kg, males: 2.48 ± 0.78 kg, *P* = 0.005).

**Table 2:** Associating prenatal or perinatal factors with calprotectin in meconium using multivariable linear or nonlinear regression analysis (stepwise, *N* = 134).

**Variables**	**B ± SE (mg/kg)**	***P*-value**	**95% Confidence interval**	
			**Lower**	**Upper**
Birth weight, per kg increase	−284.245 ± 91.881	0.002	−466.035	−102.455
Meconium-stained AF	464.696 ± 232.143	0.047	5.395	923.996

*Dependent variable was fecal calprotectin within the first week. Abbreviation: SE, standard error; meconium-stained AF, meconium-stained amniotic fluid*.

### Changes of FC and Associated Clinical Factors

We performed univariable analyses using LMM for repeated FC outcomes among 64 neonates with two or more consecutive FCs. FC decreased after 1 week of life, and the associating factors with decreased change of FC during neonatal period were males (−350.786 ± 130.211 mg/kg, *P* = 0.008), antibiotics (−429.308 ± 1,358.823 mg/kg, *P* = 0.002), and BM (−382.927 ± 149.532 mg/kg, *P* = 0.011; Table 3). GA, BW, or obstetric problems did not affect changes in FC during the neonatal period. RDS, jaundice, or intestinal distress including suspected and definite NEC did not show statistically significant effect on changes in FC during the neonatal period (Table 3). When we performed multivariable GLMM of the significantly associating factors on univariable analyses with intercept, BM (−337.266 ± 150.505, *P* = 0.026), antibiotics (−410.904 ± 141.688, *P* = 0.004), and postnatal week (second week; −416.386 ± 138.002, *P* = 0.003, and third–fourth weeks: −464.934 ± 158.024, *P* = 0.004) were associated factors with decreased changes of FC during the neonatal period compared with each reference (Figure 2 and Table 4).

**Table 3:** Changes of fecal calprotectin in neonates and associated clinical factors using univariable linear mixed analyses for repeated fecal calprotectin outcomes among 64 neonates with two or more consecutive fecal calprotectins.

**Variables**	**Estimate ± SE, mg/kg**	***P*-values**	**95% Confidence interval**	
			**Lower**	**Upper**
GA, per week increase	−23.624 ± 17.361	0.176	−57.913	10.665
BW, per kg increase	−137.669 ± 94.232	0.146	−328.777	48.439
Male^*^, female (reference)	−350.786 ± 130.211	0.008	−607.952	−93.619
5′-AS	−74.505 ± 43.771	0.091	−160.953	11.942
CS delivery, VD (reference)	−42.635 ± 157.049	0.786	−352.805	267.536
Multigestation, single (reference)	143.410 ± 139.563	0.306	−132.227	419.046
Meconium-stained AF, no (reference)	315.144 ± 209.351	0.134	−98.324	728.611
Maternal hypertensive disorder, no (reference)	−182.278 ± 140.070	0.195	−458.916	94.361
PPROM, no (reference)	306.066 ± 180.297	0.092	−50.019	662.151
GDM, no (reference)	−183.757 ± 184.587	0.321	−548.315	180.802
RDS, no (reference)	161.469 ± 132.739	0.226	−100.715	423.653
Jaundice, no (reference)	185.719 ± 144.642	0.201	−99.948	471.387
Antibiotics^*^, no (reference)	−429.308 ± 135.823	0.002	−697.559	−161.057
Intestinal distress, no (reference)	161.875 ± 148.166	0.276	−130.751	454.501
BM^*^, FM (reference)	−382.927 ± 149.532	0.011	−678.252	−87.603
Days till full feeding (120 ml/kg/day), per day increase	1.625 ± 3.687	0.660	−5.661	8.910
Hospital stays, per day increase	1.207 ± 2.075	0.562	−2.891	5.305
Mortality, no (reference)	−259.97 ± 372.944	0.487	−996.537	476.590
Postnatal week^*^, first (reference)				
Second	−427.870 ± 146.240	0.012	−781.731	−74.008
Third and fourth	−416.265 ± 164.270	0.037	−813.755	−18.774

*SE, standard error; GA, gestational age; BW, birth weight; 5′-AS, Apgar score at 5 min; CS, cesarean section; PPROM, prolonged rupture of membrane; GDM, gestational diabetes mellitus; RDS, respiratory distress syndrome; BM, breast milk; FM, formula milk*.

* *Variables were statistically significant factors associated with changes of fecal calprotectin during neonatal period on univariable analyses*.

**Table 4:** Associating clinical factor with changes of fecal calprotectin among 64 neonates with two or more consecutive fecal calprotectins by multivariable analysis using generalized linear mixed effect model for repeated calprotectin outcomes.

**Variables**	**Estimate ± SE, mg/kg**	***P*-values**	**95% Confidence interval**	
			**Lower**	**Upper**
Intercept	711.055 ± 409.573	0.085	−98.178	1, 520.289
BW, per kg increase	12.489 ± 103.234	0.904	−191.481	216.459
Male, female (reference)	−214.437 ± 128.449	0.097	−468.226	39.352
5′-AS	−7.660 ± 46.070	0.868	−98.686	83.366
BM, FM (reference)	−337.266 ± 150.505	0.026	−634.633	−39.899
Antibiotics, no (reference)	−410.904 ± 141.688	0.004	−690.851	−130.956
Intestinal distress, no (reference)	185.541 ± 163.982	0.260	−138.455	509.537
Postnatal week, first (reference)				
Second	−427.870 ± 146.240	0.012	−781.731	−74.008
Third and fourth	−464.934 ± 158.024	0.004	−777.157	−152.710

*BW, birth weight; 5′-AS, Apgar score at 5 min; BM, breast milk; FM, formula milk*.

## Discussion

Neonatal FC showed wide ranges from 5.5 to more than 6,000 mg/kg of stool in this study. Individual FC changed dynamically during the neonatal period (Figure 1), and its changes were affected by time, kind of diet, and use of antibiotics (Figure 2 and Table 4).

The authors observed that the level of FC from the first-week stool was higher than that of later FCs. The first FC in this study could reflect meconial calprotectin since it was measured on mean 2.14 ± 1.87 days after birth and preterm and term neonates could pass meconium for about 2.0–7.8 days (21). Previous studies reported that calprotectin in meconium correlated with GA at birth, BW, and poor 5′-Apgar score and that meconial calprotectin could reflect intrauterine environment, intestinal immaturity, and hypoxic–ischemic damage of the intestinal mucosa (22, 23). In our study, the high meconial calprotectin was associated with lower BW and meconium-stained AF when stepwise multivariable linear or nonlinear regression analysis was performed (Table 2). However, it was not correlated with GA (*r* = −0.125, *P* = 0.151), 5′-Apgar score (*r* = −0.057, *P* = 0.513) or postnatal days in the first week (*r* = −0.136, *P* = 0.117) unlike previous studies (22, 23). Several kinds of perinatal factors including delivery mode, feeding volume, or general conditions were reported with FC previously; however, the results were inconclusive (14, 17, 24). The lack of agreement on the results of associating perinatal factors with meconial calprotectin may have originated from diverse conditions and background in the perinatal period. Based on our results, intrauterine distress such as meconium-stained AF and intestinal immaturity in neonates with lower BW could influence high meconial calprotectin level (Table 2). However, meconium comprises materials ingested during the fetal period, including intestinal epithelial cells, AF, lanugo, and mucous. And the diverse cellular composition may affect the high concentration of meconial calprotectin because calprotectin can be expressed on the membrane of monocytes and mucosal epithelial cells besides the cytoplasm in neutrophils (2, 25).

Gender, antibiotics, diet, and postnatal week were statistically associated with change of FC, when we performed univariable analyses (Table 3). Of those significant factors, BM, antibiotics, and postnatal week were associated with decreased changes of FC during the neonatal period when multivariable analysis using GLMM with intercept was performed (Figure 2 and Table 4). In this study, a significant drop of FC was observed after the first week, and the concentration was stable after the second week of life. Resolution of intrauterine distress (23), maturation of intestinal mucosa (22), establishment of intestinal microbiota (15), or change from meconium with diverse cellular or molecular compositions to transitional stool may affect the time trend of FC between the first and second weeks of life.

FC decreased in neonates fed with BM (Tables 3 and 4, Figure 2). Selection bias may exist because meconial calprotectin of neonates fed with BM in later FCs was lower than that of neonates fed with formula milk (FM) in later FCs. However, FC increased in neonates fed with FM after the second week of life, contrary to the trend of FC in neonates fed with BM (Figure 2B). However, the influence of diet on FC is still conflicting (16, 24, 26, 27). The previous studies recruited various subjects including preterm and full-term infants aged 1 week and 5 months, and we enrolled 109 preterm and 37 full-term neonates born at 25^4/7^-40^3/7^ weeks of gestation for the neonatal period. The variation of inclusions according to the study designs may explain the conflicting results. Based on the previous report of *Bifidobacterium lactis* Bb12 and a significant decrease in calprotectin levels (12), probiotics in BM may lead to more decrease of FC than that in FM in this study. Further studies on the association between probiotics in BM and changes of FC are necessary.

Use of prenatal or perinatal antibiotic treatments could negatively or positively affect FC in newborns (15, 28). In this study, antibiotic treatment seemed to negatively affect FC during the neonatal period statistically (Figure 2C and Table 4). But selection bias existed since neonates with antibiotic treatment had already lower concentration of meconial calprotectin than neonates without antibiotic treatment in later FCs (1,404.73 ± 471.23 vs. 322.37 ± 67.72 mg/kg, *P* = 0.033). Even though there was a selection bias on statistical analysis according to antibiotic treatment in this study, the result that neonates with high meconial calprotectin did not receive antibiotics in later FCs might indirectly suggest that calprotectin may be involved in the regulation of inflammation (2).

Studies of calprotectin as a marker of intestinal disease in neonates were reported given its utility in inflammatory bowel disease (14, 17, 18, 29–33). Some of the reported FCs could be a marker for intestinal permeability such as early diagnosis and resolution of gastrointestinal illnesses (14, 15, 17, 29, 30). However, FC for the purpose of early diagnosis or prognosis of NEC has conflicting results because of wide variations of cutoff points and wide interindividual and intraindividual variations (17, 18, 33, 34). We checked FC serially during the neonatal period prospectively and reviewed intestinal distress including suspected and definite NEC via medical records, retrospectively. Suspicious NEC was analyzed together with definite NEC, because NEC is the most serious intestinal disease in neonate and suspicion is crucial. In this study, high meconial calprotectin could not expect development of intestinal distress including NEC in later FCs, and intestinal distress did not affect changes of FCs during the neonatal period (Tables 3, 4).

Our limitations were being an observational study in one hospital and enrollment of a small number of neonates with diverse clinical conditions. Thus, our results could not reflect the time trend of FC in healthy neonates. FC values tended to decrease with increasing GA or BW albeit being statistically insignificant (Table 3), which might be due to the small number of extremely preterm cases in this study (eight preterm neonates with <28 weeks of gestation and 20 stool samples). Further study with a larger number of inclusions and stool samples would be necessary because of the low reliability of this study caused by the small number of inclusions. Many missing stools at each time point and selection bias in BM and the antibiotic treatment group were the other limitations. GLMM was used for analysis between clinical factors and changes in FC to compensate for the missing samples. Despite these limitations, the prospective nature of this study and collection of serial stool samples from individuals were our strengths.

In conclusion, FC during the neonatal period decreased with time regardless of GA at birth, BW, or other diseases. BM may contribute to lower FC levels. Decrease of FC with postnatal week and BM may be associated with transition of feces or establishment of intestinal microbiota. Regarding intestinal inflammation in neonates like NEC, clinical usefulness of FC was questionable unlike children and adults. We observed dynamic changes of FC during the neonatal period, and caution is necessary when FC is interpreted in neonates.

## Data Availability Statement

All datasets generated for this study are included in the article/supplementary material.

## Ethics Statement

The studies involving human participants were reviewed and approved by the Institutional Review Board of the Gyeongsang National University Hospital. Written informed consent to participate in this study was provided by the participants' legal guardian/next of kin.

## Author Contributions

JP: Designing and conducting the study, and drafting the manuscript. JC: Conducting the study. CC: Presenting the study and collecting data. SO: Collecting data and stool samples. HD: Collecting data and stool samples. J-HS: Interpreting data. JL: Interpreting data. C-HP: Designing the study and collecting data. H-OW: Interpreting data. H-SY: Interpreting data. All authors contributed to the article and approved the submitted version.

## Conflict of Interest

The authors declare that the research was conducted in the absence of any commercial or financial relationships that could be construed as a potential conflict of interest.
